# Cannabinoid Receptor-1 suppresses M2 macrophage polarization in colorectal cancer by downregulating EGFR

**DOI:** 10.1038/s41420-022-01064-8

**Published:** 2022-05-31

**Authors:** You-Ming Deng, Cheng Zhao, Lei Wu, Zhan Qu, Xin-Yu Wang

**Affiliations:** 1 Department of Essential Surgery, Xiangya Hospital, Central South University, Changsha, 410008 Hunan Province P. R. China; 2 Department of Endocrinology, The First Affiliated Hospital of Shenzhen University, Shenzhen, 518037 Guangdong Province P. R. China; 3 Research Institute of General Surgery, Jinling Hospital, Nanjing University, Nanjing, 210093 Jiangsu Province P. R. China

**Keywords:** Diseases, Cancer

## Abstract

Cannabinoid receptors, CB1 and CB2, have been implicated as emerging targets for cancer therapy. Herein, we investigated the potential regulation mechanism of CB1 and its implications in colorectal cancer. CB1 and EGFR expression were examined in colorectal cancer cell lines. The effects of CB1 agonist ACEA and its antagonist AM251 on the proliferation, migration and invasion of colorectal cancer cells and the expression of M1 and M2 macrophage markers were examined. EGFR overexpression was performed with plasmids containing EGFR gene. Tumor xenografts were constructed to explore the effects of CB1 activation on tumorigenesis. We showed that CB1 was downregulated while EGFR was upregulated in colorectal cancer cells. The activation of CB1 suppressed the proliferation, migration and invasion of colorectal cancer cells and the differentiation of M2 macrophages, while CB1 inhibition had opposite effects. Moreover, the alterations in tumorigenesis and M2 macrophage activation induced by CB1 activation were counteracted by EGFR overexpression. Besides, CB1 silencing promoted tumor cell proliferation and M2 polarization which was counteracted by EGFR knockdown. In vivo, CB1 activation also repressed tumorigenesis and M2 macrophage activation. The present study demonstrated that CB1 activation suppressed M2 macrophage through EGFR downregulation in colorectal cancers. These findings first unveiled the potential avenue of CB1 as a targeted therapy for colorectal cancer.

## Introduction

Colorectal cancer is the fourth most common cancer worldwide, and accounts for 10.2% of the total cancer deaths [1]. The pathogenesis of colorectal cancer involves various interactions between the tumor microenvironment (TME) and the tumor cells [2]. Of all the cells that comprises TME, macrophages are major players and play a vital role in the pathogenesis of cancer by regulating tissue homeostasis and inflammation [3]. Macrophages can differentiate into pro-inflammatory M1 or anti-inflammatory M2 phenotype depending on the stimuli. Mounting evidence have suggested that M2 macrophage is essential for epithelial-mesenchymal transition (EMT) induction in cancer progression [4, 5]. For example, macrophages promoted the EMT of renal cell carcinoma by activating AKT/mTOR pathway [6]. Moreover, M2 macrophages mediated the immune evasion of cancer cells by mechanisms such as activating 15-lipoxygenase-2 pathway [7], stimulating the expression of immune regulator B7-H4 and inducing T regulatory cells, thus suppressing the antitumor response [8]. In patients with colorectal cancer, a large amount of M2 macrophages within the tumor imparts a poor prognosis [9, 10]. In vivo and in vitro studies also demonstrated that M2 macrophages promoted the migration and metastasis of colorectal cancer cells by inducing the expression of CD47 [11]. Therefore, revealing the molecular mechanisms underlying macrophage differentiation is crucial.

Cannabinoid receptors, including CB1 and CB2, are part of the endocannabinoid system. By interacting with their endogenous ligands (anandamide and 2-arachidonoylglycerol), CB1 and CB2 play vital roles in various physiological processes, including memory, pain sensation, and movement [12]. Accumulating evidence has demonstrated that cannabinoid receptors activation can suppress cancer progression in multiple tumor models [13]. In glioma, cannabinoid receptors activation-induced apoptosis and suppressed tumor growth in rats by activating ERK [14]. In lung cancer, CB2 activation suppressed EMT and tumor progression by downregulating EGFR [15]. Moreover, in colorectal cancer, cannabinoid receptors activation exerted tumour-suppressive effects in both in vitro and in vivo models [12]. However, the underlying mechanisms remain elusive.

Epidermal growth factor receptor (EGFR) is a member of the Human Epidermal Growth Factor Receptor (HER) family and plays a central role in tumorigenesis [16]. A number of studies have established that EGFR was crucial for the development of colorectal cancer [17]. In 65–75% patients with advanced colorectal cancer, EGFR is overexpressed [18], and EGFR-targeting therapies, such as cetuximab, have improved outcomes for colorectal cancer patients [19]. Several reports have indicated that the clinical benefits of EGFR-targeting agents may be mediated not only by direct suppression on the growth of tumor cells, but only by regulation on the tumor microenvironment [20]. For example, EGFR monoclonal antibody suppressed tumor metastasis by activating T cells [21]. Ravi et al. reported that EGFR mediated CB2 activation caused EMT suppression in lung cancer [15]. However, the potential link between CB1 and EGFR remains unknown.

In our present study, we aimed to explore the effect of CB1 activation on macrophage differentiation in colorectal cancer. In particular, we investigated our hypothesis that EGFR was involved in the tumor suppression and macrophage differentiation induction effects of CB1 activation. Both in vitro and in vivo experiments were carried out to test our hypothesis. Our results demonstrated that CB1 activation suppressed M2 macrophage differentiation and tumor growth by downregulating EGFR. Thus, our study suggested that CB1 may be a new strategy to suppress EGFR, and serve as a novel target for colorectal cancer treatment.

## Results

### CB1 was downregulated while EGFR was upregulated in colorectal cancer cells

**Fig. 1:**
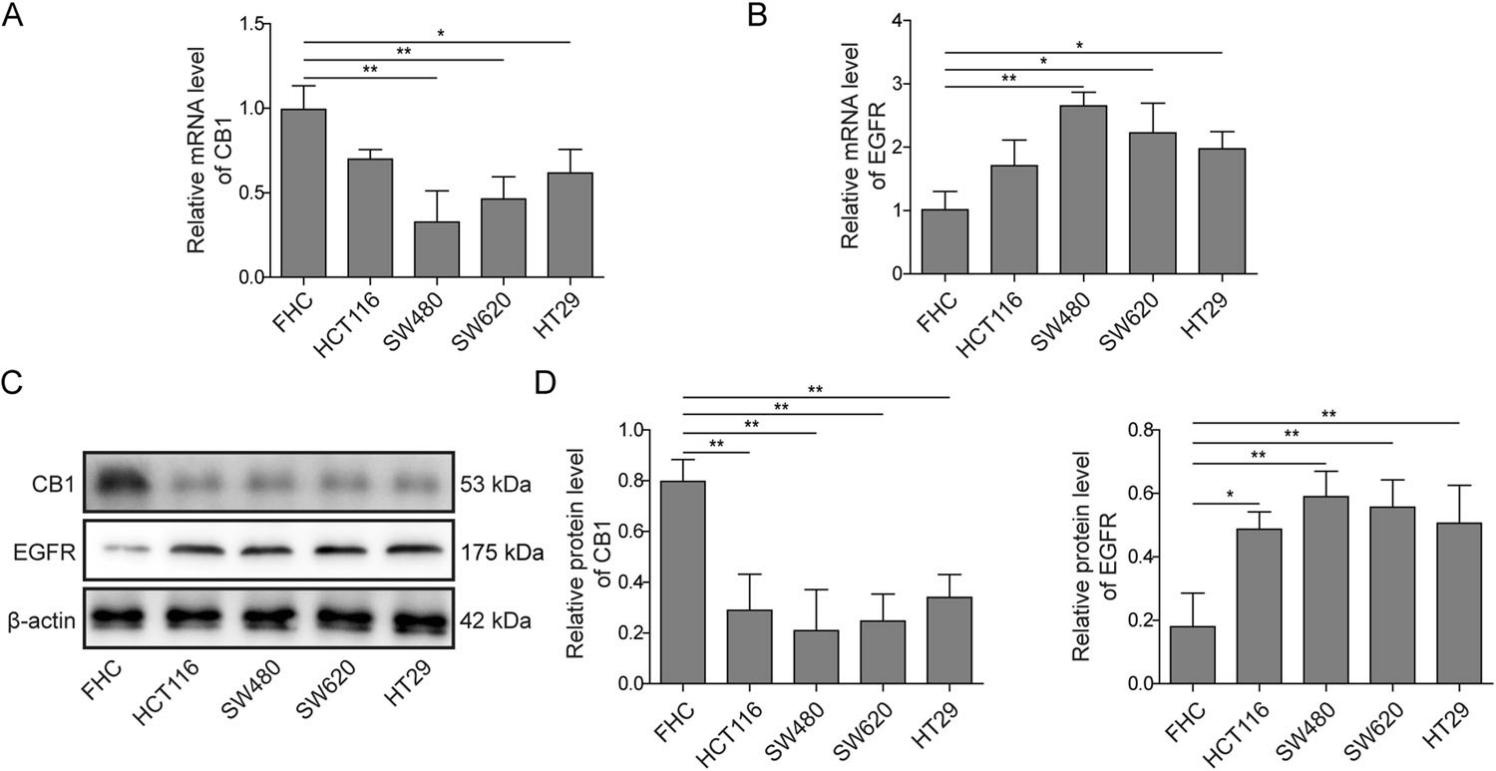
Expression of CB1 and EGFR in normal colon cells and colorectal cancer cells. **A** QPCR results showed that CB1 was downregulated in colorectal cancer cells. **B** QPCR results showed that EGFR was upregulated in colorectal cancer cells. **C, D** Western blot analysis showed that CB1 was downregulated and EGFR was upregulated in colorectal cancer cells. *P < 0.05; **P < 0.01.

Though it is well established that CB1 plays an important role in gastrointestinal functions [22, 23], little has been known about the expression of CB1 in normal and colorectal cancer cells. Hence, our study investigated the expression of CB1 in normal colon epithelial cells (FHC cells) and colorectal cancer cells (HCT116, SW480, SW620, and HT29 cells). Since we aimed to explore the potential interaction between CB1 and EGFR, we also investigated the expression of EGFR. As shown in Fig. 1A, B, compared with FHC cells, the mRNA levels of CB1 were downregulated in colorectal cancer cells, while the mRNA levels of EGFR were upregulated in colorectal cancer cells. Western blot results further validated the downregulation of CB1 and the upregulation of EGFR in colorectal cancer cells (Fig. 1C, D). According to the expression of CB1, SW480 and SW620 cells were selected for further experiments.

Taken together, these results demonstrated that CB1 was lower expressed while EGFR was higher expressed in colorectal cancer cells.

### CB1 activation repressed the proliferation, migration and invasion of colorectal cancer cells

**Fig. 2:**
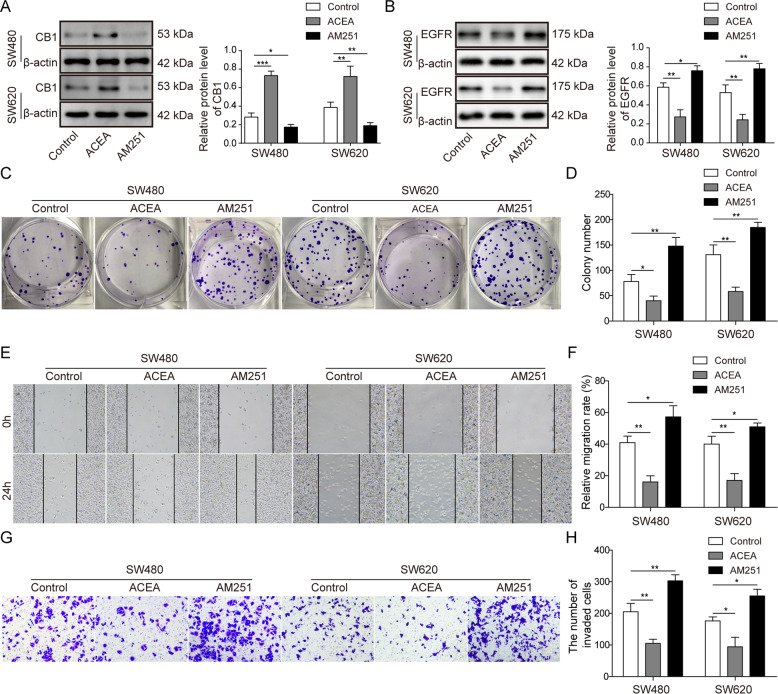
CB1 activation suppressed the proliferation, migration and invasion of colorectal cancer cells. **A** Western blot assay tested the protein level of CB1 after ACEA and AM251 treatment. **B** Western blot analysis showed that CB1 activation significantly decreased the expression of EGFR while its inhibition increased the expression of EGFR. **C, D** Colony forming assay showed that CB1 activation suppressed colony formation of colorectal cancer cells, while its inhibition promoted colony formation. **E, F** Wound healing test showed that CB1 activation prevented cell migration while CB1 inhibition enhanced cell migration of colorectal cancer cells. **G, H** Transwell invasion assay demonstrated that CB1 activation blocked cell invasion while CB1 inhibition enhanced cell invasion of colorectal cancer cells. *P < 0.05; **P < 0.01; ***P < 0.001.

We investigated the functionality of CB1 in the cell growth of colorectal cancer cells using the CB1 agonist ACEA and its antagonist AM251. After treated with ACEA, the expression of CB1 was increased significant, and AM251 inhibited the expression of CB1 (Fig. 2A). The impact of ACEA on CB2 expression was also explored. As shown in Fig. S1, ACEA treatment did not alter the expression of CB2 in both SW4280 and SW620 cells. Then, we tested the influence of CB1 activation or inhibition on EGFR expression. As shown in Fig. 2B, in both SW480 and SW620 cells, CB1 activation significantly decreased the expression of EGFR while CB1 inhibition markedly increased the expression of EGFR. Moreover, CB1 activation significantly reduced clone formation, while its inhibition remarkably enhanced clone formation of colorectal cancer cells (Fig. 2C, D). Wound healing assay results also demonstrated that CB1 activation suppressed cell migration in both cells, while CB1 inhibition had opposite effects (Fig. 2E, F). Transwell assay results showed that CB1 activation attenuated cell invasion while CB1 inhibition promoted cell invasion in colorectal cancer cells (Fig. 2G, H).

Taken together, these data supported that CB1 activation inhibited the proliferation, migration and invasion of colorectal cancer cells.

### CB1 activation inhibited M2 macrophage polarization

**Fig. 3:**
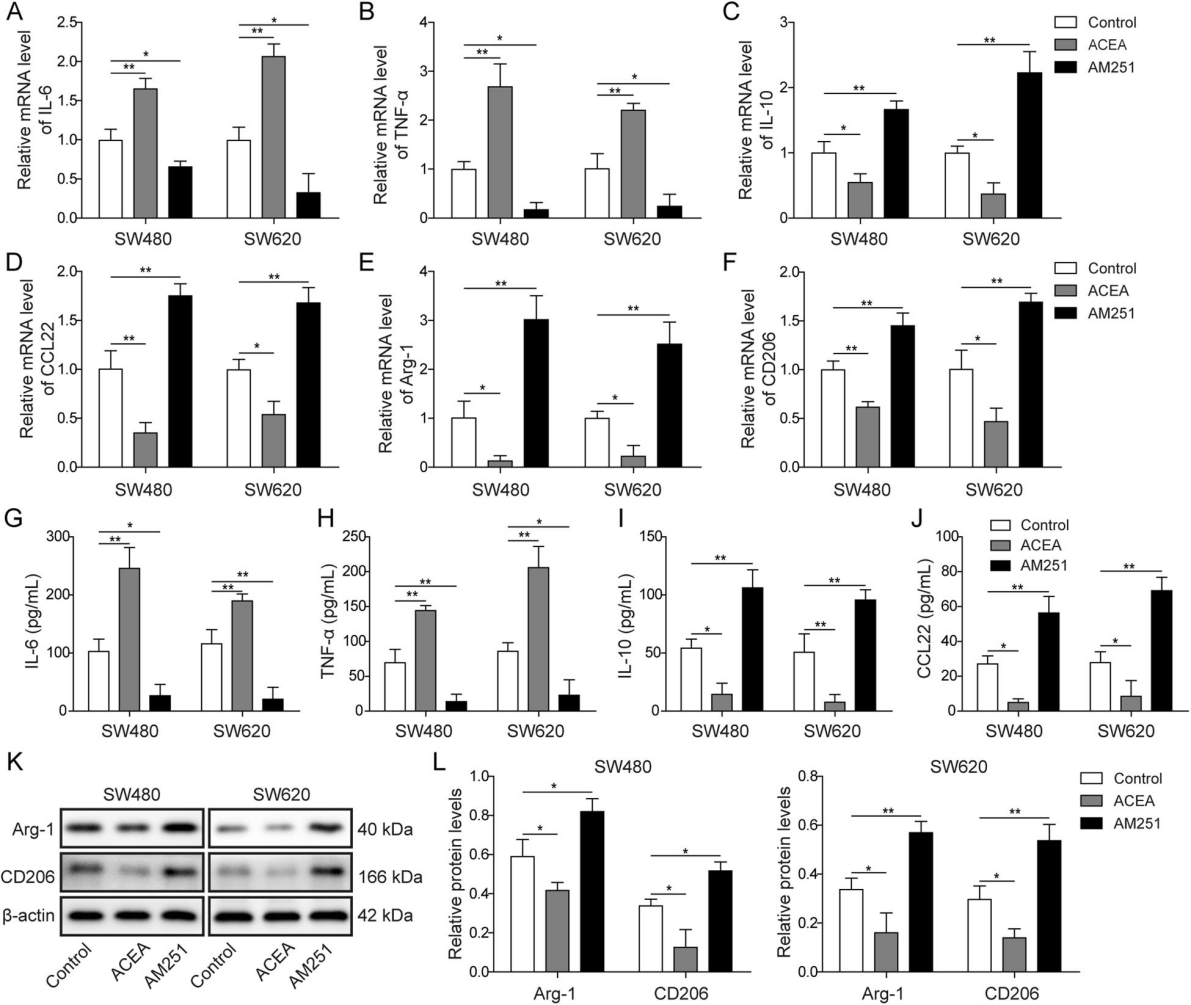
CB1 activation suppressed M2 macrophage polarization. **A, B** QPCR results showed that CB1 activation promoted the expression of M1 markers IL-6 and TNF-α, while CB1 inhibition suppressed the expression of M1 markers. **C**–**F** QPCR results showed that CB1 activation suppressed the expression of M2 markers IL-10, CCL22, Arg-1 and CD206, while CB1 inhibition promoted the expression of M2 markers. **G**–**J** ELISA results demonstrated that CB1 activation increased the release of IL-6 and TNF-α, and decreased the release of IL-10 and CCL22, while CB1 inhibition had opposite effects. **K**, **L** Western blot results showed that CB1 activation decreased the expression of Arg-1 and CD206, while CB1 inhibition increased the expression of Arg-1 and CD206. *P < 0.05; **P < 0.01.

To investigate the regulatory effect of CB1 on macrophage activation, we treated human monocyte cell THP-1 with its agonist ACEA and its antagonist AM251. Then PMA induced THP-1 cells were co-cultured with the culture medium of SW480 and SW620 colorectal cancer cells. QPCR results showed that, in cells treated with ACEA, the expression of M1 markers, including IL-6 and TNF-α, were upregulated, while M2 markers, including IL-10, CCL22, Arg-1 and CD206 were downregulated (Fig. 3A–F). In cells treated with AM251, the expression of M1 markers were downregulated, while the expression of M2 makers were upregulated (Fig. 3A–F). ELISA assay was also employed to measure the cytokines in the culture medium. As shown in Fig. 3 G–J, in ACEA-treated group, IL-6 and TNF-α were upregulated, and IL-10 and CCL22 were downregulated; in AM251-treated group, IL-6 and TNF-α were downregulated, and IL-10 and CCL22 were upregulated. Moreover, western blot assay results also showed that CB1 activation decreased the expression of Arg-1 and CD206 while CB1 inhibition elevated the expression of Arg-1 and CD206 (Fig. 3K, L).

Taken together, these results demonstrated that CB1 activation suppressed M2 macrophage polarization.

### CB1 suppressed cancer cell growth by downregulating EGFR

**Fig. 4:**
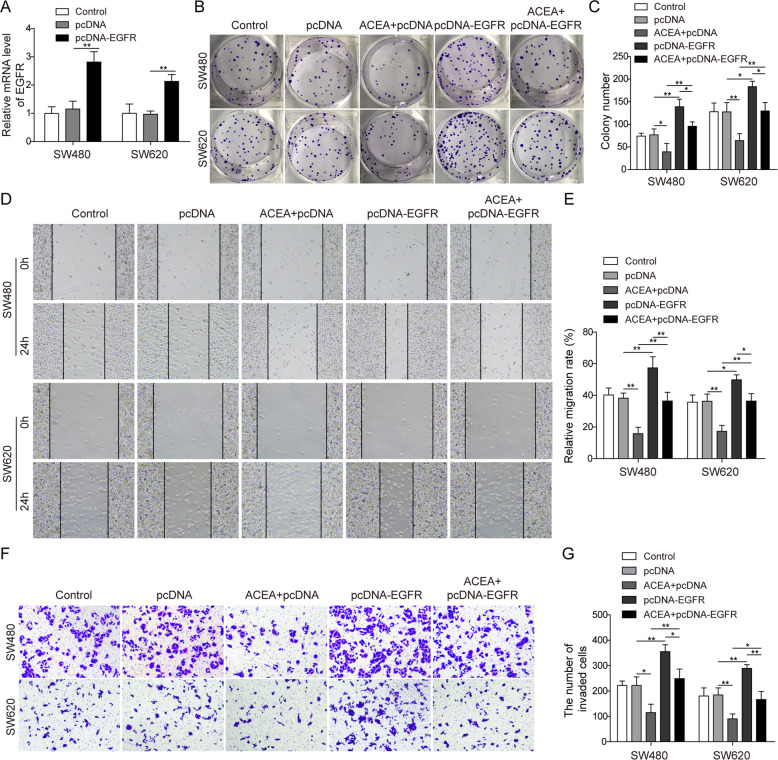
EGFR was involved in the antitumor effects of CB1 activation. Cells were transfected with EGFR plasmids and simultaneously treated with ACEA. **A** QPCR analysis showed that EGFR plasmids transfection increased the expression of EGFR in SW480 and SW620 cells. **B**, **C** Colony-forming assay demonstrated that EGFR overexpression blocked the proliferation suppression induced by CB1 activation. **D**, **E** Wound healing test showed that EGFR overexpression counteracted the migration inhibition induced by CB1 activation. **F**, **G** Transwell invasion assay showed that EGFR overexpression prevented the decrease in cell invasion caused by CB1 activation. *P < 0.05; **P < 0.01.

Since EGFR was reported to mediate antitumor effect of CB2 activation [15], we explored whether EGFR was involved in the antitumor effect of CB1 in colorectal cancer. EGFR was overexpressed in SW480 and SW620 cells using EGFR plasmids, and the cells treated with ACEA simultaneously. The level of EGFR in transfected cells was significantly upregulated by QPCR (Fig. 4A). Results from colony formation, wound healing assay and transwell invasion tests consistently demonstrated that EGFR overexpression completely blocked CB1-activation-induced suppression in cell proliferation, migration and invasion in colorectal cancer cells (Fig. 4B–G).

Taken together, these data demonstrated that EGFR overexpression completely reversed CB1 activation-induced tumor suppression.

### CB1 inhibited M2 macrophage polarization by downregulating EGFR

**Fig. 5:**
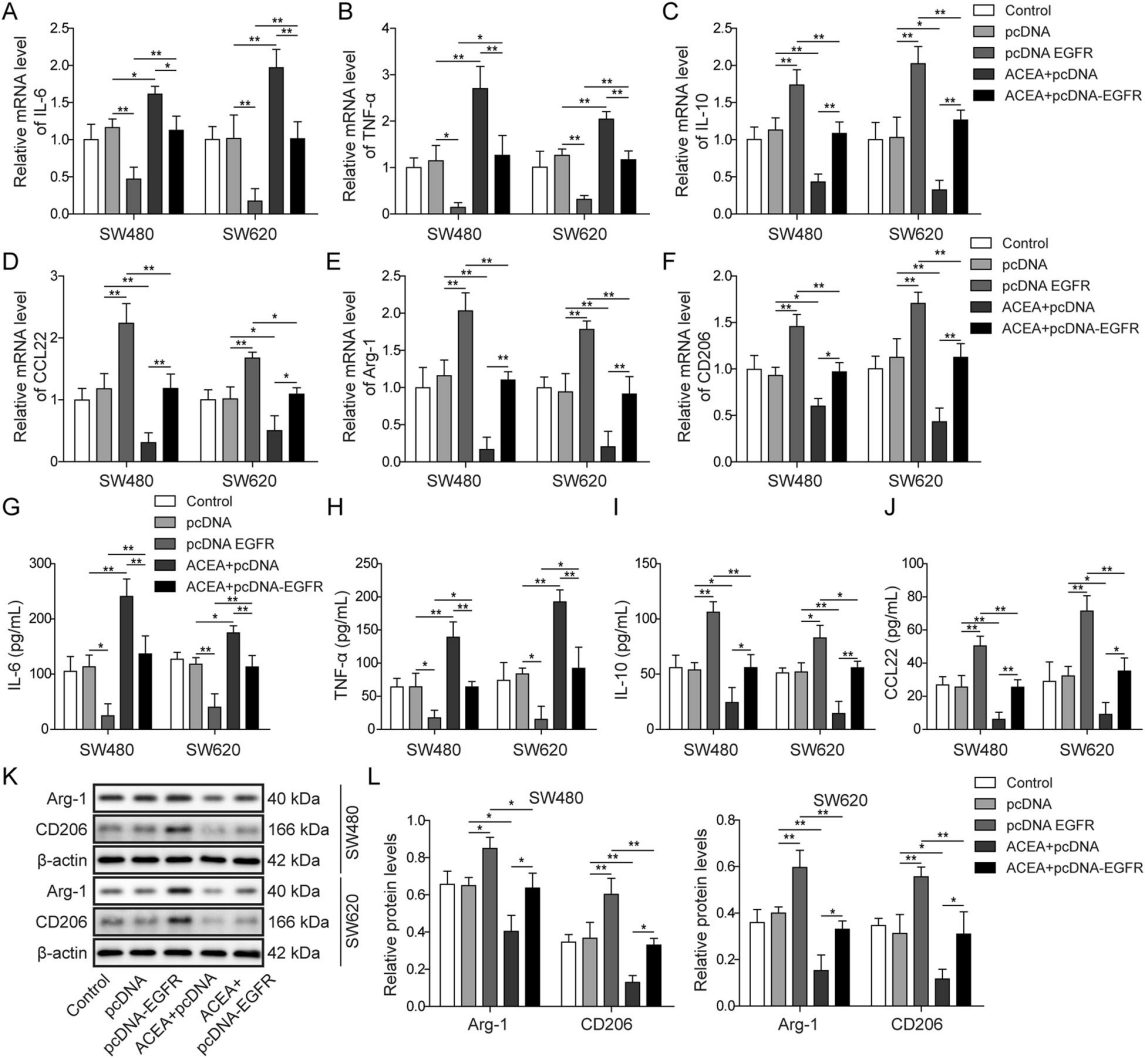
CB1 activation suppressed M2 macrophage polarization by regulating EGFR. Colorectal cancer cells were transfected with EGFR plasmids and simultaneously treated with ACEA. Then PMA induced THP-1 cells were co-cultured with the culture medium of colorectal cancer cells. **A**–**F** QPCR results demonstrated that EGFR overexpression blocked CB1 activation caused increase in the expression of IL-6 and TNF-α, and decrease in IL-10, CCL22, Arg-1 and CD206. **G**–**J** ELISA assay showed that EGFR overexpression blocked CB1 activation caused increase in the release of IL-6 and TNF-α, and decrease in the release of IL-10 and CCL22. **K**, **L** Western blot showed that EGFR overexpression prevented the decrease in the expression of Arg-1 and CD206. *P < 0.05; **P < 0.01.

To explore whether EGFR mediated the effects of CB1 on macrophage polarization, we overexpressed EFGR and co-treated with ACEA in colorectal cancer cells. Then the PMA-induced THP-1 cells were incubated with the culture medium collected from colorectal cancer cells. We found that EGFR overexpression totally prevented CB1 activation induced changes in the expression of IL-6, TNF-α, IL-10, CCL22, Arg-1 and CD206 (Fig. 5A–F). Moreover, ELISA assay results showed that the changes in the release of IL-6, TNF-α, IL-10 and CCL22 induced by CB1 activation were also reversed by EGFR overexpression (Fig. 5G–J). Besides, the decreasing protein levels of Arg-1 and CD206 caused by ACEA were restored by EGFR overexpression (Fig. 5K, L).

Taken together, these findings indicated that EGFR overexpression completely reversed CB1 activation-induced inhibition of M2 macrophage polarization.

### EGFR knockdown reversed the effects of CB1 silencing on tumor cell growth and M2 macrophage polarization

**Fig. 6:**
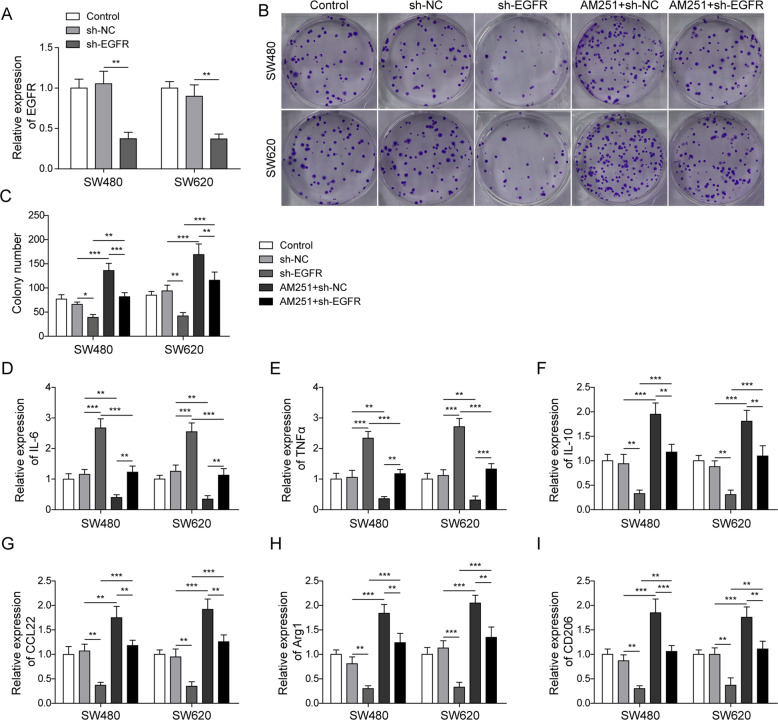
EGFR knockdown reversed the effects of CB1 silencing on tumor cell growth and M2 macrophage polarization. Colorectal cells were transfected with plasmids bearing shRNA against EGFR and simultaneously treated with AM251. Culture medium for these colorectal cells were taken to incubate PMA-induced THP-1 cells. **A** QPCR analysis showed that sh-EGFR effectively suppressed the expression of EGFR. **B**, **C** Colony formation test demonstrated that EGFR knockdown reversed the enhancement in colony formation induced by AM251. **D**–**I** QPCR results demonstrated that EGFR knockdown reversed the CB1 inhibition caused decrease in the expression of IL-6 and TNF-α, and increase in IL-10, CCL22, Arg-1 and CD206. *P < 0.05; **P < 0.01; ***P < 0.001.

To further confirm the effect of CB1 on EGFR mediated macrophage polarization, we knocked down EFGR and co-treated with AM251 in colorectal cancer cells. As shown in Fig. 6A, sh-EFGR effectively suppressed the expression of EGFR. Colony formation assay result showed that EGFR knockdown significantly suppressed colony formation while CB1 silencing remarkably increased colony formation, and EGFR silencing abolished the promotion on colony formation in AM251 treated cells (Fig. 6B and C). The effect of CB1 silencing and EGFR knockdown was also investigated with PMA-induced THP-1 cells. These THP-1 cells were incubated with culture media from SW480 and SW620 for 24 hours and the expression of M1 and M2 polarization markers was determined. As shown in Fig. 6D–I, EGFR knockdown significantly up-regulated IL-6 and TNF-α, while down-regulated IL-10, CCL22, Arg-1and CD206, whereas CB1 silencing exerted opposite effects. What’s more, EGFR knockdown reversed the effects of CB1 inhibition on the expression of macrophage polarization markers in cells treated with media from colorectal cells (Figs. 6D–I). Taken together, these results highlighted the essential role of CB1-EGFR axis in colorectal cancer cell proliferation and macrophage M2 polarization.

### CB1 activation suppressed tumorigenesis in vivo

**Fig. 7:**
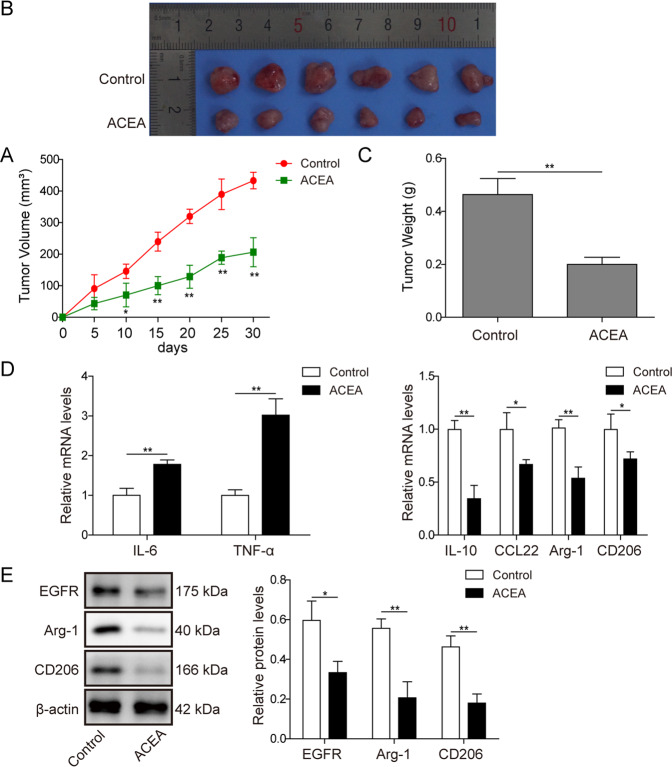
CB1 activation suppressed tumorigenesis in vivo. SW480 cells were subcutaneously injected into nude mice and the tumors were treated with control or ACEA (1.5 mg/kg/d). **A** CB1 activation suppressed tumor growth. Representative tumors (**B**) and tumor weight (**C**) from different experimental groups. **D** QPCR analysis showed that CB1 activation increased the expression of IL-6 and TNF-α, and decreased the expression of IL-10, CCL-22, Arg-1 and CD206. **E** Western blot analysis showed that the expression of EGFR, Arg-1 and CD206 were decreased in the tumors of ACEA-treated mice. *P < 0.05; **P < 0.01.

To assess the antitumor effect of CB1 activation, we constructed an in vivo model by subcutaneously injecting SW480 cells into the right flank of nude mice. Then we treated the tumors with ACEA, tumor volumes were measured every 5 days. As shown in Fig. 7A, ACEA administration remarkably slowed the growth of tumor cells. Moreover, ACEA administration also significantly decreased the tumor size and weight (Fig. 7B, C). Besides, the expression of IL-6 and TNF-α were upregulated while the expression of IL-10, CCL22, Arg-1 and CD206 were downregulated in the tumor tissues from ACEA-treated group (Fig. 7D). Western blot results also demonstrated that ACEA administration significantly decreased the expression of EGFR, Arg-1 and CD206 (Fig. 7E).

Taken together, these results suggested that ACEA suppressed tumorigenesis and M2 macrophage differentiation in vivo.

## Discussion

Mounting evidence demonstrates that TME plays a crucial role in tumor initiation, progression and evolution [24]. Macrophages, as a critical regulator of TME, play a central role in tumor progression, immune evasion and chemoresistance [25]. However, the mechanisms underlying macrophage differentiation regulation remains poorly understood. A recent study revealed that, CB2 activation blocked M2 macrophage differentiation [15]. Given the functional and structural similarity between CB1 and CB2, it is plausible to assume that CB1 is also capable to regulate macrophage polarization. However, there has been few reports regarding this problem. In the present study, we revealed the regulatory effect of CB1 activation on macrophage differentiation and the underlying mechanisms.

Initially, we found that CB1 was downregulated in colorectal cancer cells, which was consistent with the findings of Cianchi et al. [26]. Our results also showed that EGFR was overexpressed in colorectal cancer cells, which is consistent with the fact that EGFR overexpression has been observed in about 70% colorectal cancer patients [17]. A previous report has suggested that CB1 activation suppressed tumor development by inducing apoptosis in colorectal cancer cells [23]. ACEA is an endogenous cannabinoid analog and a selective cannabinoid CB1 receptor agonist, and AM251 is a selective CB1 antagonist [27]. In our study, ACEA and AM251 were employed to explore the potential role of CB1 in colorectal cancer. Our study showed that ACEA and AM251 could not only activate/inactivate CB1, and also regulate the expression of CB1. A compensatory mechanism might underlie the regulation of these agonist and antagonist on CB1 expression. In addition, we found that ACEA treatment did not impact the expression of CB2 in both SW480 and SW620 cells, excluding the possibility that the changes observed with ACEA might be caused by CB2. In line with our study, similar observations have been reported by other teams [28–31], and related mechanisms included enhancing promoter activity via Akt and NF-kB or inducing Human antigen R nucleoplasmic transport. Our results also showed that CB1 activation inhibited the proliferation, migration and invasion of colorectal cancer cells. Moreover, CB1 activation significantly suppressed M2 macrophage differentiation, which was confirmed by decrease in M2 markers IL-10, CCL22, Arg-1 and CD206. To our knowledge, this is the first report suggesting the regulatory role of CB1 on M2 macrophage differentiation. Since M2 macrophages are reported to promote carcinogenesis via immune suppression and EMT promotion [6], the tumor suppressive of CB1 activation can be at least partly attributed to its regulation on macrophage differentiation. This is of great significance since the protumor function of macrophages has been shown in various cancers [32]. Thus, CB1 can be used as a target to modulate macrophage differentiation in many cancers.

Our study also found the underlying mechanism by which CB1 activation repressed M2 macrophage differentiation and tumor growth. The regulation of CB1 on EGFR is not surprising, since several reports have documented the regulation of CB2 on EGFR [15, 33]. Moreover, our results confirmed the link between EGFR and macrophage differentiation, since EGFR overexpression significantly promoted M2 macrophage activation [34, 35]. Our findings are strengthened by other reports, in which EGFR knockout suppressed M2 macrophage differentiation and carcinogenesis in colorectal cancer [36]; in addition, Zhang et al. also reported that EGFR inhibition markedly decreased M2 activation in colorectal cancer [37]. Through enhancing EGFR expression, we proved that EGFR mediated ACEA-caused changes in tumor growth and macrophage differentiation. We also found that EGFR knockdown suppressed colony formation and macrophage M2 polarization, while promoted macrophage M1 polarization; CB1 silencing had opposite effects which were all blocked by co-transfection with EGFR shRNA. Together with these findings, our study highlighted the crucial role of CB1 as a negative regulator of EGFR to modulate macrophage activation.

Since in vivo studies are important to validate the findings from in vitro experiments, we further confirmed our results in tumor xenografts of SW480 cells in nude mice. Consistent with our in vitro results, we found that CB1 activation suppressed tumor growth in tumor xenografts of colorectal cancer. Moreover, CB1 activation also downregulated the expression of IL-10, CCL22, Arg-1 and CD206, suggesting that CB1 activation suppressed M2 activation. Due to the pro-tumorigenic effects of M2 macrophages, we postulated that the tumour-suppressive effects of CB1 activation in vivo could be ascribed to its effect on macrophage differentiation. As a preliminary study, the present study has several limitations. First, the mechanism how AM251 and ACEA regulated the expression of CB1 was not explored, and future studies are necessary. Second, the interaction between CB1 and EGFR was not discovered in the present study. The interplay between cannabinoid receptors and EGFR has been reported by multiple groups [15, 38, 39], while the mechanisms remain elusive. Fiori et al. reported that inactivation of CB1 with AM251 upregulated EGFR by destabilizing oestrogen-related receptor alpha in four human cancer cells which expresses very low levels of CB1 [40]. Apart from this paper, few studies have revealed related molecular details. This interplay would be one of our future focuses.

In conclusion, our study showed that CB1 activation suppressed tumor growth and M2 macrophage activation in colorectal cancer by downregulating EGFR. Our study provided the first evidence that CB1 activation was capable to suppress M2 macrophage activation. Since M2 macrophage are linked with immune evasion in various cancers, CB1 might be a promising target for cancer treatment.

## Materials and methods

### Cell culture

Fetal human colon cell line FHC, human colorectal cancer cell lines, HCT116, SW480, SW620 and HT29, and human monocytic cell line THP-1 was purchased from ATCC (Manassas, VA, USA). THP-1 cells and FHC cells were cultured in RPMI-1640 (Hyclone, Logan, UT, USA) while other cells were grown in DMEM (Hyclone). The culture medium was supplemented with 10% heat-inactivated FBS (Gibco, Carlsbad, CA, USA) and 1% penicillin/streptomycin (Gibco). SW480 and SW620 cells were treated with 1 μM ACEA or 10 μM AM251 for 24 h and then used for further experiments.

THP-1 cells were treated with 320 nM phorbol 12-myristate 13-acetate (PMA, Sigma-Aldrich, St. Louis, MO, USA) for 24 h to induce differentiation into M0 macrophages. Then the cells were incubated with the culture medium collected from colorectal cancer cells for another 48 h.

### Cell transfection

Plasmids for EGFR and EGFR shRNA (sh-EGFR) were purchased from Thermofisher (San Jose, CA, USA). Plasmids were transfected into SW480 and SW620 cells using Lipofectamine 3000 (Invitrogen, Shanghai, China). After 48 h, cells were prepared for further experiments. The expression of EGFR and CB1 in the transfected cells were examined by qRT-PCR assay.

### Colony-forming assay

2000 cells were seeded in to each well and treated with vehicle, ACEA (1 μM) or AM251 (10 μM) for 14 days. After washing with PBS, cells were stained with 0.1% crystal violet and clones were observed by a light microscope and counted.

### Wound healing test

1 × 10^5^ cells were seeded into each well of a 24-well culture plate. When the cells reached 80% confluency, the monolayer cells were scratched vertically with a sterile pipette tip. At 0 and 24 h, the cells were photographed with a microscope in four random areas. Cells in the gap were analyzed with GraphPad.

### Transwell invasion assay

Cell invasion was determined with the Transwell system (Corning, NY, USA). The transwells were precoated with Matrigel (100 μg/cm^2^, BD Biosciences, San Jose, CA, USA) in dH_2_O overnight. Then 5 × 10^4^ cells were seeded in each well and subjected to drug treatment or plasmid transfection or both for 48 h. Subsequently, the cells were stained with 0.1% crystal violet and visualized with a microscope.

### ELISA assay

The expression of IL-6, TNF-α, IL-10 and CCL22 in the culture medium were determined with a commercial Elisa kit (Thermofisher) according to the manufacturer’s instructions.

### QRT-PCR

Total RNA was isolated with Trizol (Sigma Aldrich) and reverse-transcribed into cDNA using cDNA Reverse Transcription Kit (Life Technologies, Carlsbad, CA, USA). The qRT-PCR was performed using a SYBR-Green system (Life Technologies). All primers were purchased from Sangon (Shanghai, China), and listed as follows:

CB1, forward, 5’-AGGAGTAAGGACCTGCGACA-3’ and reverse, 5’-TCTTGACCGTGCTCTTGATG-3’;

EGFR, forward, 5’-GGTCTTGAAGGCTGTCCAACG-3’ and reverse, 5’-CCTCAAGAGAGCTTGGTTGGG-3’;

IL-6, forward, 5’-ACAGGGAGAGGGAGCGATAA-3’ and reverse, 5’-GAGAAGGCAACTGGACCGAA-3’;

TNF-α, forward, 5’-CCCCAGGGACCTCTCTCTAA-3’ and reverse, 5’-TGAGGTACAGGCCCTCTGAT-3’;

IL-10, forward, 5’-GCCAAGCCTTGTCTGAGATG-3’ and reverse, 5’-GGCCTTGCTCTTGTTTTCAC-3’;

CCL22, forward, 5’-ATGGATCGCCTACAGACTGC-3’ and reverse, 5’-CGGCACAGATCTCCTTATCC-3’;

Arg-1, forward, 5’-CCAAGGTCTGTGGGAAAAGCA-3’ and reverse, 5’-TACAGGGAGTCACCCAGGAG-3’;

CD206, forward, 5’-ACTAGGCAATGCCAATGGAG-3’ and reverse, 5’-TGGTCAGCGGGTCTTTATTC-3’;

GAPDH was used as internal control, forward, 5’-CCAGGTGGTCTCCTCTGA-3’ and reverse, 5’-GCTGTAGCCAAATCGTTGT-3’.

### Western blot analysis

Cells or tissues were lysed with RIPA buffer (Thermofisher), centrifuged, and supernatant were collected as protein samples. Equal amounts of protein were loaded, separated by a 10% SDS-PAGE gel, and then transferred onto NC membranes. After blocking with 5% BSA, membranes were incubated with primary antibodies: Arg-1, 93668, CST (Boston, Massachusetts, USA); CD206, ab64693, Abcam (Cambridge, MA, USA); CB1, ab259323 Abcam; CB2, ab3561 Abcam; EGFR, ab52894, Abcam; β-actin, ab8226, Abcam at 4 °C overnight. Subsequently, the membranes were incubated with HRP-conjugated secondary antibody at room temperature for 1 h. Then signals were then detected with an ECL detection kit (Thermofisher).

### In vivo tumorigenicity assay

All animal procedures were approved and supervised by the Ethics Committee of Xiangya Hospital of Central South University.12 8-week-old male immunodeficient nude mice were brought from SLRC (Shanghai, China). The mice were subcutaneously injected with 1 × 10^6^ SW480 cells, and randomly divided into 2 groups: the control group and the ACEA group. 5 days later, the ACEA group was injected peritumorally with ACEA (1.5 mg/Kg/d) while the control group received vehicle in PBS. Tumor volumes were measured every 5 days. After one month, the mice were killed.

### Statistical analyses

All data are presented as mean ± SD. Each experiment was repeated at least 3 times with duplicates. For single comparison, Student’s t test was used; for multiple comparisons, one-way ANOVA, followed by Student-Newman-Keuls post hoc test was used. A *p* value less than 0.05 were considered statistically significant.

## Supplementary information

Supplementary information

FigS1

Original Data File

## Data Availability

All data generated or analyzed during this study are included in this article. The datasets used and/or analyzed during the current study are available from the corresponding author on reasonable request.

## References

[1] Bray F, Ferlay J, Soerjomataram I, Siegel RL, Torre LA, Jemal A (2018). Global cancer statistics 2018: GLOBOCAN estimates of incidence and mortality worldwide for 36 cancers in 185 countries. CA: a Cancer J Clin.

[2] Llosa NJ, Cruise M, Tam A, Wicks EC, Hechenbleikner EM, Taube JM (2015). The vigorous immune microenvironment of microsatellite instable colon cancer is balanced by multiple counter-inhibitory checkpoints. Cancer Discov.

[3] Erreni M, Mantovani A, Allavena P (2011). Tumor-associated macrophages (TAM) and inflammation in colorectal cancer. Cancer Microenviron.

[4] Liu C-Y, Xu J-Y, Shi X-Y, Huang W, Ruan T-Y, Xie P (2013). M2-polarized tumor-associated macrophages promoted epithelial–mesenchymal transition in pancreatic cancer cells, partially through TLR4/IL-10 signaling pathway. Lab Investig.

[5] She L, Qin Y, Wang J, Liu C, Zhu G, Li G (2018). Tumor-associated macrophages derived CCL18 promotes metastasis in squamous cell carcinoma of the head and neck. Cancer Cell Int.

[6] Yang Z, Xie H, He D, Li L (2016). Infiltrating macrophages increase RCC epithelial mesenchymal transition (EMT) and stem cell-like populations via AKT and mTOR signaling. Oncotarget.

[7] Daurkin I, Eruslanov E, Stoffs T, Perrin GQ, Algood C, Gilbert SM (2011). Tumor-associated macrophages mediate immunosuppression in the renal cancer microenvironment by activating the 15-lipoxygenase-2 pathway. Cancer Res.

[8] Chen S, Zhang JQ, Chen JZ, Chen HX, Qiu FN, Yan ML (2017). The over expression of long non-coding RNA ANRIL promotes epithelial-mesenchymal transition by activating the ATM-E2F1 signaling pathway in pancreatic cancer: An in vivo and in vitro study. Int J Biol Macromol.

[9] Herrera M, Herrera A, Dominguez G, Silva J, Garcia V, Garcia JM (2013). Cancer-associated fibroblast and M2 macrophage markers together predict outcome in colorectal cancer patients. Cancer Sci.

[10] Algars A, Irjala H, Vaittinen S, Huhtinen H, Sundstrom J, Salmi M (2012). Type and location of tumor-infiltrating macrophages and lymphatic vessels predict survival of colorectal cancer patients. Int J Cancer.

[11] Zhang Y, Sime W, Juhas M, Sjolander A (2013). Crosstalk between colon cancer cells and macrophages via inflammatory mediators and CD47 promotes tumour cell migration. Eur J Cancer.

[12] Di Marzo V, Piscitelli F (2015). The Endocannabinoid System and its Modulation by Phytocannabinoids. Neurotherapeutics: J Am Soc Exp NeuroTherapeutics..

[13] Chakravarti B, Ravi J, Ganju RK (2014). Cannabinoids as therapeutic agents in cancer: current status and future implications. Oncotarget.

[14] Galve-Roperh I, Sanchez C, Cortes ML, Gomez del Pulgar T, Izquierdo M, Guzman M (2000). Anti-tumoral action of cannabinoids: involvement of sustained ceramide accumulation and extracellular signal-regulated kinase activation. Nat Med.

[15] Ravi J, Elbaz M, Wani NA, Nasser MW, Ganju RK (2016). Cannabinoid receptor‐2 agonist inhibits macrophage induced EMT in non‐small cell lung cancer by downregulation of EGFR pathway. Mol Carcinogenesis.

[16] Seshacharyulu P, Ponnusamy MP, Haridas D, Jain M, Ganti AK, Batra SK (2012). Targeting the EGFR signaling pathway in cancer therapy. Expert Opin Therapeutic Targets.

[17] Krasinskas AM (2011). EGFR Signaling in Colorectal Carcinoma. Pathol Res Int.

[18] Lockhart AC, Berlin JD (2005). The epidermal growth factor receptor as a target for colorectal cancer therapy. Semin Oncol.

[19] de Castro-Carpeno J, Belda-Iniesta C, Casado Saenz E, Hernandez Agudo E, Feliu Batlle J, Gonzalez Baron M (2008). EGFR and colon cancer: a clinical view. Clin Transl Oncol.

[20] Ferris RL, Jaffee EM, Ferrone S (2010). Tumor antigen-targeted, monoclonal antibody-based immunotherapy: clinical response, cellular immunity, and immunoescape. J Clin Oncol.

[21] Garrido G, Lorenzano P, Sanchez B, Beausoleil I, Alonso DF, Perez R (2007). T cells are crucial for the anti-metastatic effect of anti-epidermal growth factor receptor antibodies. Cancer Immunol, immunotherapy: CII.

[22] Di Francesco A, Falconi A, Di Germanio C, Di Bonaventura MVM, Costa A, Caramuta S (2015). Extravirgin olive oil up-regulates CB1 tumor suppressor gene in human colon cancer cells and in rat colon via epigenetic mechanisms. J Nutritional Biochem.

[23] Messerini L, Manera C, Ronconi E, Romagnani P, Donnini M, Perigli G (2008). Cannabinoid Receptor Activation Induces Apoptosis through Tumor Necrosis Factor A^ Mediated Ceramide De novo Synthesis in Colon Cancer Cells. Clin Cancer Res.

[24] Chen F, Zhuang X, Lin L, Yu P, Wang Y, Shi Y (2015). New horizons in tumor microenvironment biology: challenges and opportunities. BMC Med.

[25] Chanmee T, Ontong P, Konno K, Itano N (2014). Tumor-associated macrophages as major players in the tumor microenvironment. Cancers..

[26] Cianchi F, Papucci L, Schiavone N, Lulli M, Magnelli L, Vinci MC (2008). Cannabinoid receptor activation induces apoptosis through tumor necrosis factor alpha-mediated ceramide de novo synthesis in colon cancer cells. Clin Cancer Res.

[27] Pertwee RG Pharmacological actions of cannabinoids. Handbook of experimental pharmacology. 2005;168:1–51. 10.1007/3-540-26573-2_1 16596770

[28] Yang S, Hu B, Wang Z, Zhang C, Jiao H, Mao Z (2020). Cannabinoid CB1 receptor agonist ACEA alleviates brain ischemia/reperfusion injury via CB1–Drp1 pathway. Cell Death Discov.

[29] Wang S, Li B, Shen X, Duan H, Guo Z, Li X (2021). The cannabinoid receptor CB1 affects the proliferation and apoptosis of adenomyotic human uterine smooth muscle cells of the junctional zone: a mechanism study. Reprod Biol Endocrinol.

[30] Laprairie RB, Kelly MEM, Denovan-Wright EM (2013). Cannabinoids increase type 1 cannabinoid receptor expression in a cell culture model of striatal neurons: Implications for Huntington’s disease. Neuropharmacology.

[31] Chang N, Duan X, Zhao Z, Tian L, Ji X, Yang L (2020). Both HuR and miR-29s regulate expression of CB1 involved in infiltration of bone marrow monocyte/macrophage in chronic liver injury. J Cell Physiol.

[32] Mantovani A, Marchesi F, Malesci A, Laghi L, Allavena P (2017). Tumour-associated macrophages as treatment targets in oncology. Nat Rev Clin Oncol.

[33] Elbaz M, Ahirwar D, Ravi J, Nasser MW, Ganju RK (2017). Novel role of cannabinoid receptor 2 in inhibiting EGF/EGFR and IGF-I/IGF-IR pathways in breast cancer. Oncotarget..

[34] Hardbower DM, Singh K, Asim M, Verriere TG, Olivares-Villagomez D, Barry DP (2016). EGFR regulates macrophage activation and function in bacterial infection. J Clin Investig.

[35] Lian G, Chen S, Ouyang M, Li F, Chen L, Yang J. Colon Cancer Cell Secretes EGF to Promote M2 Polarization of TAM Through EGFR/PI3K/AKT/mTOR Pathway. Technol Cancer Res Treat. 2019;18:1533033819849068. 10.1177/1533033819849068 PMC6535704 31088266

[36] Hardbower DM, Coburn LA, Asim M, Singh K, Sierra JC, Barry DP (2017). EGFR-mediated macrophage activation promotes colitis-associated tumorigenesis. Oncogene..

[37] Zhang W, Chen L, Ma K, Zhao Y, Liu X, Wang Y (2016). Polarization of macrophages in the tumor microenvironment is influenced by EGFR signaling within colon cancer cells. Oncotarget..

[38] Fowler CJ, Hammarsten P, Bergh A (2010). Tumour Cannabinoid CB(1) receptor and phosphorylated epidermal growth factor receptor expression are additive prognostic markers for prostate cancer. PLoS One.

[39] Yang H, Wang Z, Capó-Aponte JE, Zhang F, Pan Z, Reinach PS (2010). Epidermal growth factor receptor transactivation by the cannabinoid receptor (CB1) and transient receptor potential vanilloid 1 (TRPV1) induces differential responses in corneal epithelial cells. Exp Eye Res.

[40] Fiori JL, Sanghvi M, O’Connell MP, Krzysik-Walker SM, Moaddel R, Bernier M (2011). The cannabinoid receptor inverse agonist AM251 regulates the expression of the EGF receptor and its ligands via destabilization of oestrogen-related receptor alpha protein. Br J Pharmacol.

