# Supplementary material for: Cannabinoid Receptor-1 suppresses M2 macrophage polarization in colorectal cancer by downregulating EGFR
Source: Cell Death Discov. 2022 May 31;8:273. doi: 10.1038/s41420-022-01064-8 (PMC9156763; doi:10.1038/s41420-022-01064-8)
Supplement: Supplementary file 1 — Supplementary information [file 41420_2022_1064_MOESM1_ESM.docx]

**Supplementary information Figure Legend**

**Figure S1.** CB2 expression was not impacted by ACEA. Both SW480 and SW620 cells were treated with 1 μM ACEA. (A, B) Western blot analysis found that the expression of CB2 was not impacted by ACEA treatment.
