# Supplementary figures and images for: Cannabinoid Receptor-1 suppresses M2 macrophage polarization in colorectal cancer by downregulating EGFR
Source: Cell Death Discov. 2022 May 31;8:273. doi: 10.1038/s41420-022-01064-8 (PMC9156763; doi:10.1038/s41420-022-01064-8)

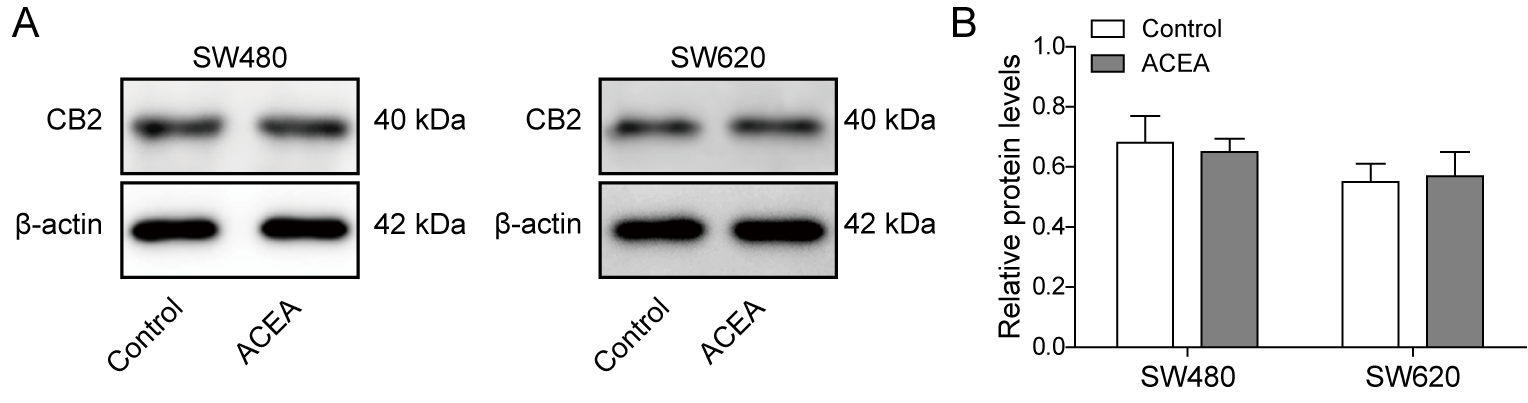

Supplement: Supplementary file 2 — FigS1 [file 41420_2022_1064_MOESM2_ESM.tif]

Original WB images

Fig1C


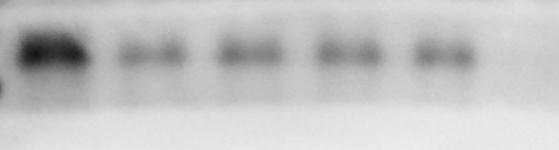

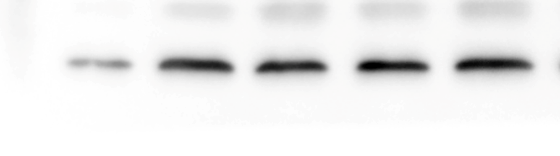


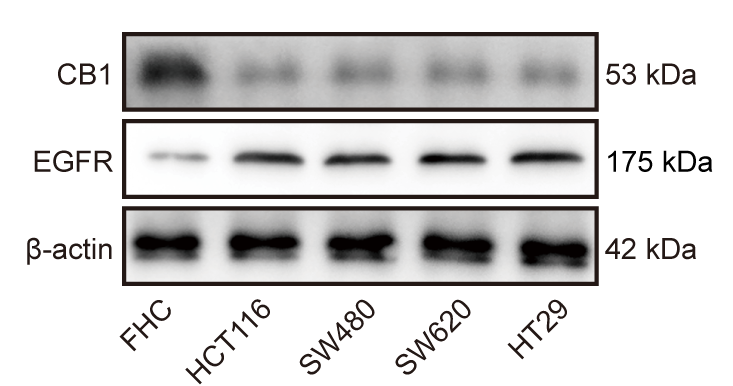


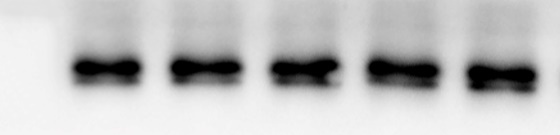


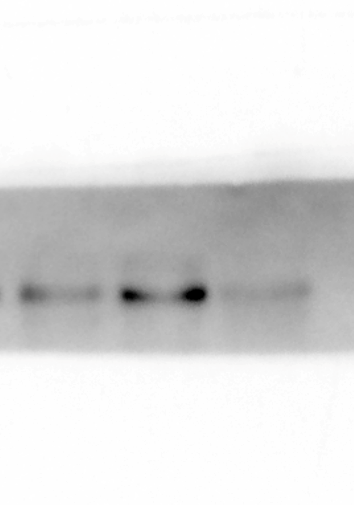


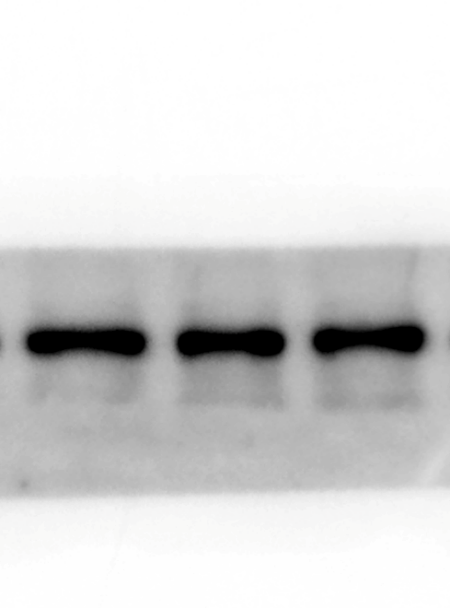


Fig2A


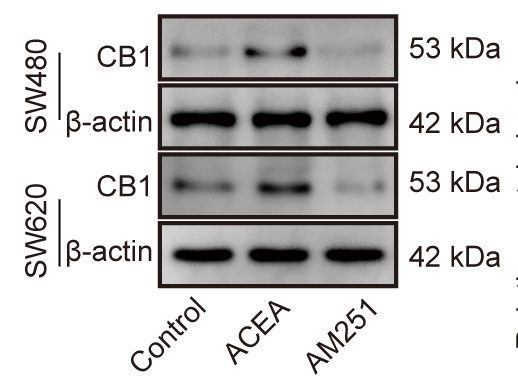


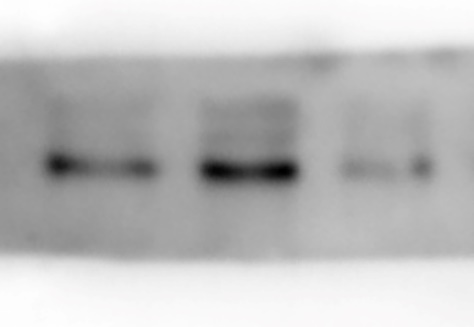


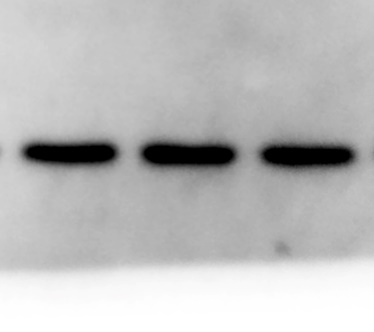


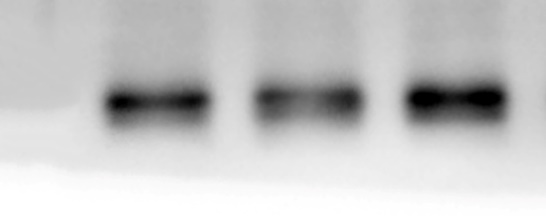


Fig2B


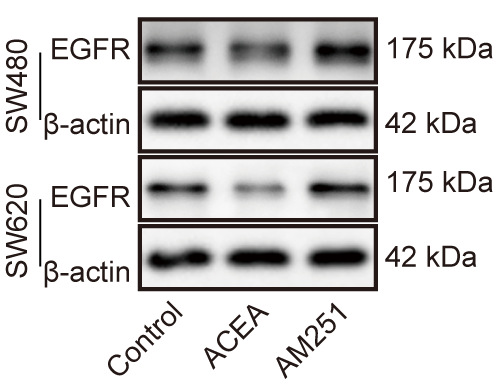


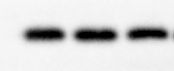


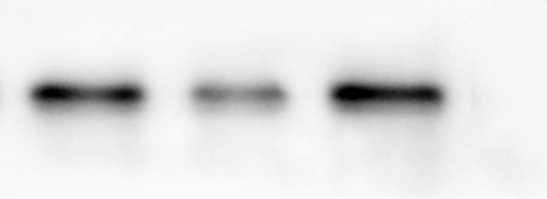


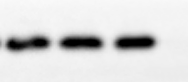


Fig3K


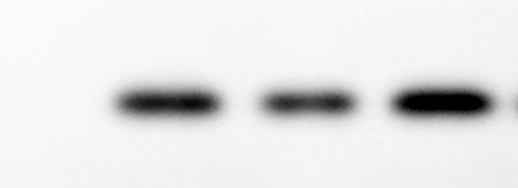

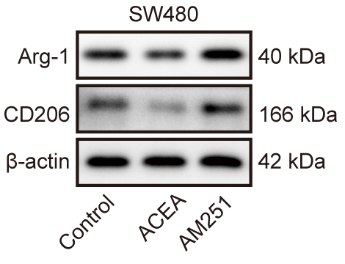

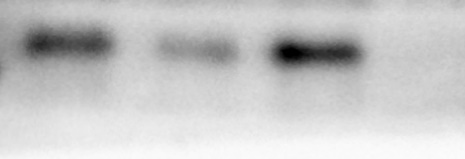

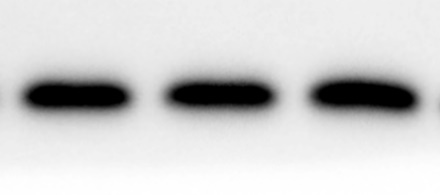


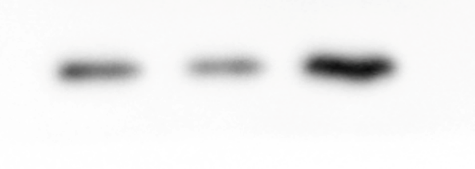


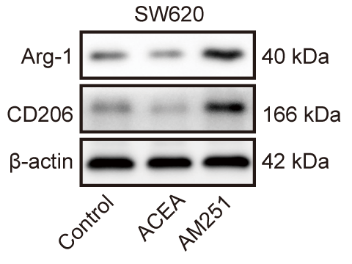


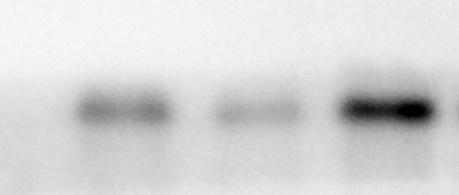


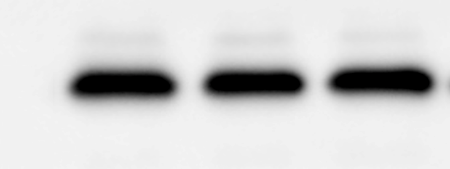


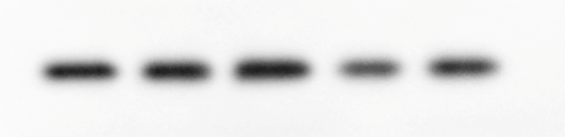


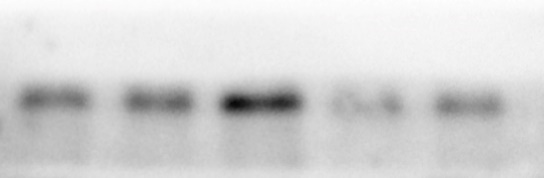


Fig5K


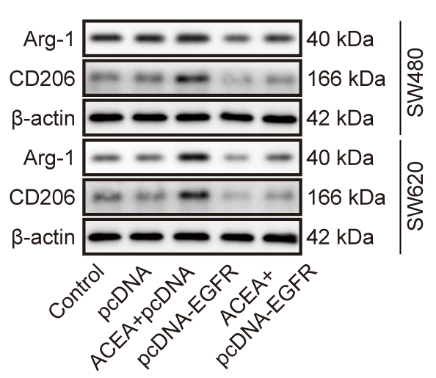


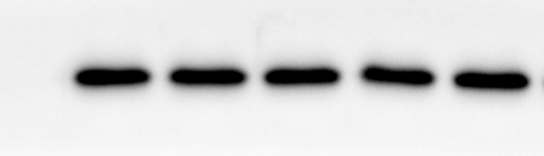


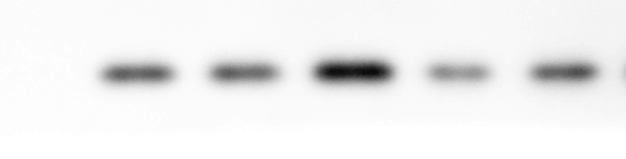


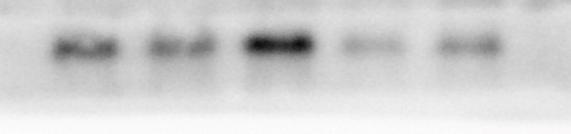


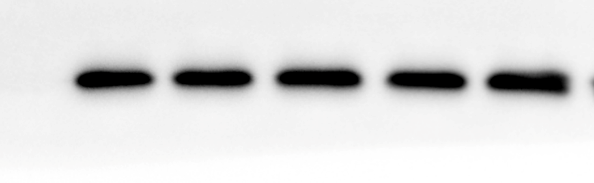


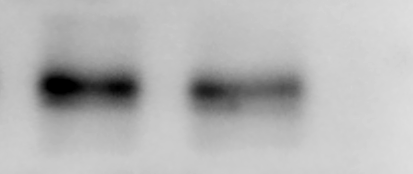


Fi**g7E**


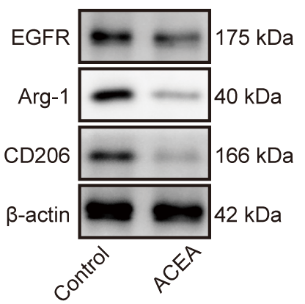

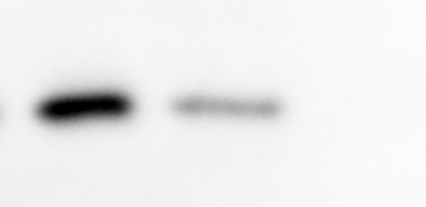

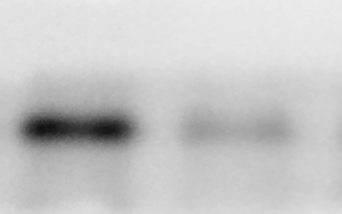

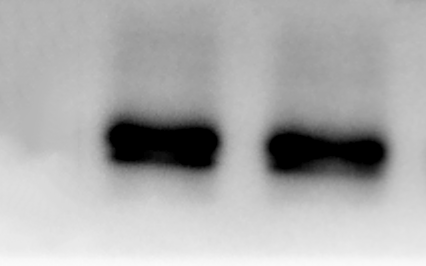


**FigS1A**


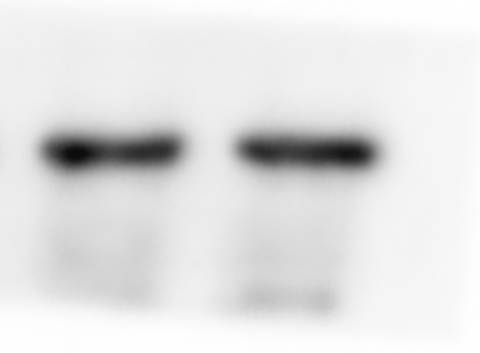


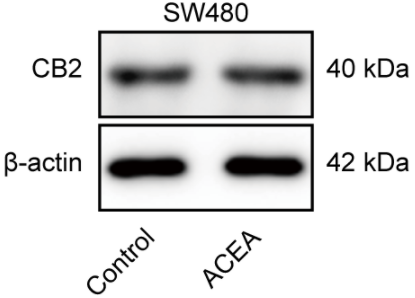


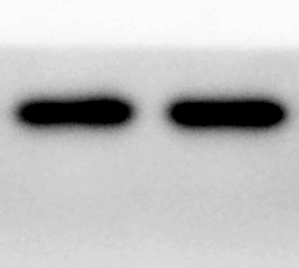

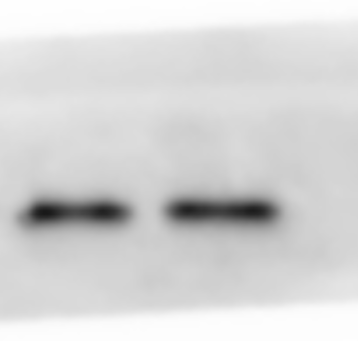

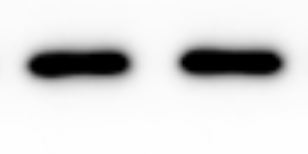

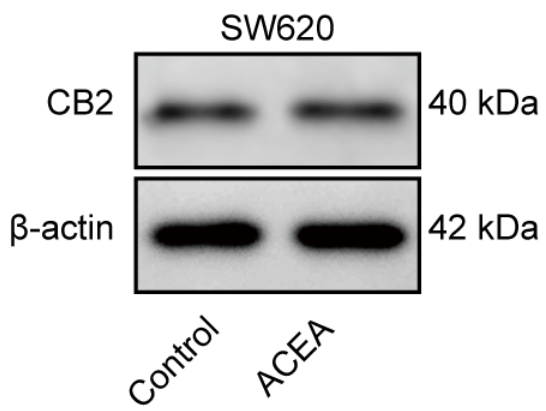

Supplement: Supplementary file 3 — Original Data File [file 41420_2022_1064_MOESM3_ESM.docx]
